# Integrated transcriptomic and immune-associated network analysis of breast cancer patient-derived organoids reveals candidate inflammatory biomarkers

**DOI:** 10.3389/fbioe.2026.1900123

**Published:** 2026-08-28

**Authors:** S. Scippa, A. Balestrieri, M. Zanfardino, M. Salvatore, A. Soricelli, M. D’aiuto, G. Mossetti, V. Ciaramella

**Affiliations:** 1 IRCCS SYNLAB SDN, Naples, Italy; 2 Department of Medical, Movement and Well-being Sciences, University of Naples Parthenope, Naples, Italy; 3 Clinica Villa Fiorita, Aversa, Italy; 4 Pathological Anatomy Service, Maria Rosaria Clinic, Pompei/Naples, Italy

**Keywords:** biomarker discovery, breast cancer, differential gene expression, inflammation targeting, patient-derived organoids

## Abstract

**Background:**

Despite advances in targeted therapies, breast cancer remains one of the major challenges to global health. Although patient-derived organoids (PDOs) are physiologically relevant models, existing transcriptomic studies are limited by the poor integration of immune signals and the absence of shared biomarkers across subtypes. In this study, we hypothesised that an integrated transcriptomic and network-based analysis of PDOs could identify conserved transcriptional signatures and pathway interactions across breast cancer subtypes.

**Materials and methods:**

PDOs representative of multiple breast cancer subtypes were analysed and compared with non-tumour organoids. Differential gene expression analysis was performed to identify transcriptional alterations, followed by pathway enrichment analysis and protein-protein interaction network analysis to investigate functional pathways and molecular interactions. Candidate biomarkers identified through computational analyses were subsequently validated experimentally by assessing gene expression and cytokine secretion profiles in tumour PDOs.

**Results:**

A total of 646 differentially expressed genes were identified. Pathway enrichment analysis revealed translational machinery and ribosome biogenesis as the dominant statistically significant processes, consistent with hyperactivated protein synthesis in cancer cells. Although neurotrophin signaling pathways were not enriched at the transcriptome-wide level, network analysis identified a computationally predicted functional association between IL1RAP and NTRK3 in the STRING database. SLITRK3, IL1RAP and IRF2BP2 were consistently overexpressed in tumour PDOs and associated with activation of inflammatory pathways and increased secretion of IL-1 and IFN-γ. NTRK3 was identified as a direct network interactor of IL1RAP bridging inflammatory and neurotrophin signalling, though its transcriptional direction in BC PDOs could not be unambiguously resolved from the available data. Experimental validation confirmed the upregulation of SLITRK3, IL1RAP and IRF2BP2 together with elevated secretion of IL-1β and IFN-γ.

**Discussion:**

Our findings suggest that IL1RAP and IRF2BP2 may be important in immune-related and candidated biomarker identification in breast cancer PDOs. We integrated transcriptomic, enrichment and network-based analyses for a robust strategy for biomarker discovery. This methodologically transparent framework highlights potential targets in breast cancer. Rather than only a list of candidate genes, it provides an organoid-derived computational framework for risk stratification.

## Graphical Abstract

**Figure:**
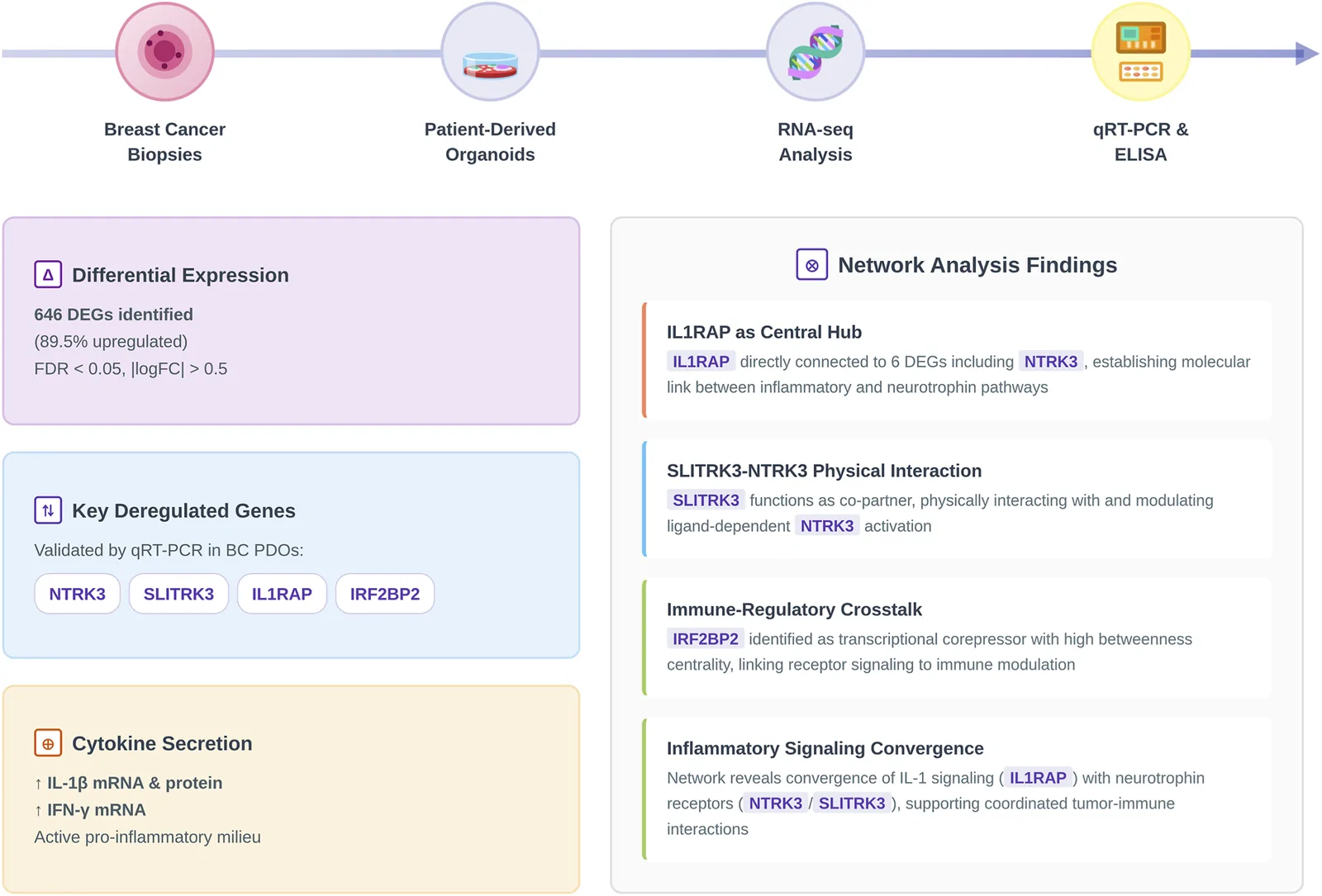
Integrative transcriptomic and immune profiling in breast cancer patient-derived organoids. Patient-derived organoids (PDOs) were established from breast cancer biopsies and subjected to RNA-seq analysis, followed by qRT-PCR and ELISA validation. Differential expression analysis identified 646 differentially expressed genes (DEGs), of which 89.5% were upregulated. Four key deregulated genes (NTRK3, SLITRK3, IL1RAP, and IRF2BP2) were validated by qRT-PCR in BC PDOs compared to non-cancerous controls. Functional validation demonstrated elevated secretion of pro-inflammatory cytokines IL-1β (mRNA and protein) and IFN-γ (mRNA), confirming an active inflammatory microenvironment. Protein-protein interaction network analysis revealed IL1RAP as a central hub directly connected to 6 DEGs including NTRK3, establishing a molecular link between inflammatory and neurotrophin signaling pathways. SLITRK3 was identified as a physical co-partner modulating NTRK3 activation, while IRF2BP2 emerged as a transcriptional corepressor linking receptor signaling to immune modulation. These findings highlight the convergence of IL-1 signaling with neurotrophin receptors, supporting coordinated tumor-immune interactions in breast cancer PDOs.

## Introduction

Despite significant advances in the understanding of breast cancer biology and the development of improved therapeutic strategies over recent decades, this malignancy remains a major global public health challenge. In 2022, approximately 2.3 million women were diagnosed with breast cancer worldwide, and both incidence and mortality rates continue to rise globally, with an expected increase of 38% new cases and 68% deaths by 2050, disproportionately affecting low- and middle-income countries (Kim et al., 2025). Nevertheless, these trends exhibit considerable geographic variation, influenced by differences in risk factor prevalence, implementation of screening programs, and access to modern therapeutic approaches.

Over the past decades, the identification of numerous molecular markers has enhanced the biological characterization and clinical management of breast cancer. These include immunohistochemical markers such as estrogen receptor (ER), progesterone receptor (PR), human epidermal growth factor receptor 2 (HER2), and the proliferation marker Ki-67; genomic markers such as BRCA1 and BRCA2 mutations; and immunological markers including immune checkpoint proteins (e.g., PD-L1) and tumor-infiltrating lymphocytes (TILs). Collectively, these biomarkers are now integral to patient stratification and guide the selection of personalized treatment regimens, including emerging targeted therapies (Orrantia-Borunda, 2022). The use of patient-derived organoids (PDOs) has emerged as a powerful tool for translating the classic cancer cell line culture into a 3D patient-specific model to study cancer biology. It has been demonstrated that PDOs are more physiologically relevant due to their ability to preserve the heterogeneity and molecular characteristics that are inherent to the tissue of origin. The primary tumor is removed, and subsequent functional studies on cancer growth, drug response, and mechanisms of resistance are facilitated. This approach provides a valuable research resource and new insights into personalized medicine approaches (Della Corte et al., 2019; Guillen et al., 2022). The molecular characterization of PDOs through transcriptomic profiling has provided novel insights into gene expression patterns associated with different breast cancer subtypes. In parallel, increasing evidence highlights the pivotal role of the immune system in modulating tumor progression and therapeutic response. Chronic inflammation plays an important dual role in cancer, contributing both to tumor-promoting processes such as angiogenesis, cellular proliferation, and immune evasion, and to anti-tumor immunity. The tumor microenvironment is shaped by a complex interplay of inflammatory cytokines, immune cells, and stromal elements, which influence cancer progression and response to therapy (Psilopatis et al., 2023; Morgillo et al., 2018). Concurrently, the neurotrophin signaling pathway has been implicated in cancer cell plasticity, survival, and metastasis, with emerging evidence of its interaction with inflammatory processes (Mahdi et al., 2023; Bollig-Fischer et al., 2021). Emerging evidence highlights the importance of neurotrophin signaling in interfacing with the tumor microenvironment and modulating cancer cell plasticity, thus representing a promising target for therapeutic intervention in breast cancer (Bruno et al., 2022; Hunt et al., 2021). The crosstalk between innate inflammatory signaling and neurotrophin pathways may represent a critical interface mediating tumor growth, immune modulation, and cellular adaptability in breast cancer.

In this study, we conducted a comprehensive transcriptomic analysis of breast cancer patient-derived organoids (PDOs) aiming to characterize the functional network connecting different pathways to better understand how the integration of inflammatory and neurotrophic signals influences tumor growth, cellular plasticity, and the immune microenvironment. These insights could reveal novel subtype-specific biomarkers highlighting the utility of PDOs as a platform for precision oncology.

## Materials and methods

### Establishment of patient-derived 3D organoid cultures

A total of 20 female patients undergoing breast surgery at Clinica Villa Fiorita S.p.A. (Aversa, Italy) between April 2024 and February 2025 were enrolled in this study. Tissue samples were classified as non-cancerous (NC) when a clinical diagnosis of fibroadenoma was established, and as breast cancer (BC) when malignancy was confirmed by histopathological analysis. All procedures involving human samples were conducted in accordance with the ethical standards of the institutional and national research committees and with the 1964 Helsinki Declaration and its later amendments. Clinical-pathological characteristics of the patients were described in Table 1. The study protocol was approved by the Ethics Committee of IRCCS Pascale (Naples, Italy), under reference number 3/19, approved on 29 May 2019. Written informed consent was obtained from all participating patients and healthy donors prior to sample collection. *Ex vivo* 3D organoid cultures (PDOs) were established from freshly collected breast cancer tissue samples using a previously described protocol (Della Corte et al., 2019). In brief, tumour specimens were maintained on ice and processed under sterile conditions on the same day of surgical resection. Tissue fragments were enzymatically digested in PDO culture medium supplemented with hyaluronidase and collagenase (Sigma-Aldrich) and incubated at 37 °C on a shaking platform set at low to moderate speed (approximately 200 rpm) for 12–18 h. Following digestion, cell suspensions were filtered and subjected to sequential centrifugation steps to remove debris and enrich viable cell populations. Organoid structures became visible within 48 h and were passaged by direct dilution, with the cell density being adjusted to fit the assay plate. All experiments were performed using PDOs from the first passage, in line with our previous validation which demonstrated that the PDO platform preserves the histological architecture, genetic profile and immune cell populations of the corresponding primary tumours during the first passage (Della Corte et al., 2019).

**TABLE 1:** Clinical-pathological characteristics of the selected patients.

Patient-derived organoids, n = 20			
Breast cancer (n = 10)	​	Fibroadenoma (n = 10)	
Sex	​	Sex	​
Female	10	Female	10
Male	0	Male	0
Histologic types	​	Histologic types	​
Invasive breast cancer ‘no special type’	8	Intra-canalicular	9
Invasive lobular carcinoma	2	Intra-and peri-canalicular	1
Ki67	​	-	-
Low (0%–29%)	6	-	-
High (30%–100%)	4	-	-
Grade	​	-	-
G1	3	-	-
G2	5	-	-
G3	2	-	-
Malignant tumor size	​	Benign tumor size	​
(0.1–2 cm)	4	(0.1–2.5 cm)	3
(2–5 cm)	6	(2.5–5 cm)	7

### Immunofluorescence and flow cytometry analyses

Confocal microscopy analysis was performed to characterize PDOs. For this purpose, images were acquired after 3 days of formation. The cell nuclei were stained with Hoechst 33,342 (Thermo Scientific™, #H3570) at a final concentration of 1 μg/mL for 1 h at 37 °C. To demonstrate the morphology of the PDO organoids and the presence of lymphocytes, CD326-APC (also known as EpCAM, or Epithelial Cell Adhesion Molecule) (Beckman Coulter, #B90408) and PE-Cy™7 Mouse Anti-Human CD45 (BD Pharmingen™, #560915) were utilized in a 1:100 ratio for 1 h at 37 °C. Maximum projection images were acquired using confocal microscopy (Mica, Leica Microsystems, Wetzlar, Germany) at ×63 magnification and saved in TIFF format. After 5 days, all patients’ PDOs were analyzed using a CytoFLEX (Beckman Coulter, Brea, CA, United States). Specifically, PDOs were stained for surface marker detection using the following antibody mixture: EpCAM-APC and CD45-PE. Each antibody was prepared at a dilution of 1:10 and mixed thoroughly. All the antibodies used were purchased from Beckman Coulter. Cell characterization was performed using Kaluza 2.1 software (Beckman Coulter, United States).

### RNA isolation and qRT-PCR

Total RNA was extracted from cells and early-passage patient-derived organoids (PDOs) using 1 ml of Trizol reagent (Life Technologies). To minimize phenotypic and molecular drift and preserve the biological characteristics of the original tumors, all transcriptomic analyses were performed using early-passage PDOs. Subsequently, chloroform (Life Technologies) was added to the Trizol at a 1:5 ratio, and the mixture was centrifuged at 12,000 rpm for 10 min at 4 °C to allow phase separation. The RNA-containing phase was then collected and combined with 500 µL of isopropanol (Life Technologies), followed by centrifugation at 12,000 rpm for 10 min at 4 °C. After discarding the supernatant, the resulting pellet was washed three times with 1 mL of 70% ethanol (Life Technologies), each washing involving centrifugation at maximum speed for 5 min. Ethanol was then removed, and the pellet was left to air dry to ensure complete evaporation. The dried pellet was resuspended in RNAse-free water, and RNA concentration was measured using a NanoDrop One spectrophotometer (Thermo Fisher). For cDNA synthesis, 1 µg of isolated RNA was reverse transcribed using the SensiFAST reverse transcriptase kit (Bioline), following the manufacturer’s instructions. Quantitative real-time PCR (RT-qPCR) was performed to assess gene expression levels. Gene-specific primers were sourced from the Eurofins Genomics website and amplification was carried out using SYBR Green PCR Master Mix (Applied Biosystems). The thermal cycling conditions were as follows: an initial step at 50 °C for 2 min, followed by denaturation at 95 °C for 10 min, and 40 cycles of 95 °C for 15 s and 60 °C for 1 min. Each reaction was run in duplicate with a 20 µL final volume using a QuantStudio 7 Flex system (Applied Biosystems). Gene expression levels (primer sequences listed in (Table 2) were normalized to 18S ribosomal RNA as an internal control, and relative expression was calculated using the 2^^–ΔΔCt^ method.

**TABLE 2:** Primer sequences used for qPCR.

Gene	Forward sequence	Reverse sequence
RPS18	CGC​CGC​TAG​AGG​TGA​AAT​TC	CTT​TCG​CTC​TGG​TCC​GTC​TT
NTRK3	CCG​ACA​CTG​TGG​TCA​TTG​GCA​T	CAG​TTC​TCG​CTT​CAG​CAC​GAT​G
SLITRK3	ACA​GAC​GCC​TTT​GCT​GGC​ACA​T	TCA​CAG​GTG​CAG​TCC​CAA​GGA​T
IL-1β	GCT​GAT​GGC​CCT​AAA​CAG​ATG	TTG​CTG​TAG​TGG​TGG​TCG​GA
IFN-γ	ATG​GCT​GAA​CTG​TCG​CAA​G	TGC​AGG​CAG​GAC​AAC​CAT​T

### Enzyme linked immunosorbent assay (ELISA)

The Human IL-1β (Interleukin 1 Beta) ELISA kit (AssayGenie) was used to measure IL-1β expression in cancer cell lines and patient-derived organoids (PDOs). The assay was conducted using a pre-coated 96-well plate, with wells designated for standards, test samples and controls (zero), ensuring each condition was tested in duplicate. Before adding reagents, the plate was washed twice with Wash Buffer. Then, 100 µL of standard solution was added to the standard wells, 100 µL of Standard Dilution Buffer into the control wells and 100 µL of properly diluted Test samples—including human serum, plasma and tissue homogenates—were pipetted to the designated wells. Then, the plate was sealed with a cover and incubated at 37 °C for 90 min. After incubation, the cover was removed, the excess liquid aspirated and the plate was washed twice with Wash Buffer, without allowing the wells to dry. Subsequently, 100 µl of Biotin-labelled Antibody working solution was added to each well—standard, test sample and zero wells—without touching the side walls. The plate was then sealed again and incubated at 37 °C for 60 min. Following this incubation, the cover was removed and the plate was washed three times, allowing the Wash Buffer to remain in the wells for 1–2 min between each wash. Next, 100 µL of SABC working solution was added to each well, followed by a 30-min incubation at 37 °C. The plate was then washed five times, letting the wash buffer remain in each well for 1–2 min per wash. TMB substrate was added to each well (90 µl) for colorimetric detection. Then, the plate was incubated in the dark at 37 °C for 10 to 20 min, allowing a visible blue color to develop in the wells containing the highest concentration of standards. The reaction was stopped by adding 50 µL of Stop Solution, resulting in an immediate color change to yellow. Absorbance was then measured at 450 nm using a microplate reader.

### Differential gene expression analysis

Raw read count data were analyzed using the edgeR (v3.42.4) and limma (v3.46.0) R packages to identify differentially expressed genes (DEGs) between biological conditions. The input consisted of a non-normalized count matrix with genes as rows and samples as columns. Sample grouping was defined (n = 10 per group) to compare “BC” (breast cancer) versus “NC” (non-cancerous tissues) conditions. Genes with low expression were filtered out using the filterByExpr () function, requiring a minimum of 10 counts in at least three samples. Normalization of library sizes was performed using the Trimmed Mean of M-values (TMM) method via calcNormFactors (). A design matrix reflecting group structure was built with model.matrix (), and dispersion estimates (common, trended, and tagwise) were obtained using estimateDisp () with robust estimation. Gene-wise negative binomial generalized linear models were fitted using glmFit (), and differential expression testing was carried out with the likelihood ratio test (glmLRT ()), using a contrast that compared BC versus NC. DEGs were selected based on a false discovery rate (FDR) threshold of 0.05 and an absolute log_2_ fold change greater than 0.5. Visualization included MA plots and volcano plots to assess the distribution and significance of DEGs. To further investigate NTRK3 expression at the isoform level, transcript-level quantification was performed using Salmon (v1.10, decoy-aware index, GRCh38/GENCODE v44), followed by Differential Transcript Expression (DTE) analysis with DESeq2 and Differential Transcript Usage (DTU) analysis with DRIMSeq and stageR. In-silico PCR of the qRT-PCR primers against GENCODE v44 NTRK3 transcript sequences was performed to assess isoform specificity of the amplicon. Raw RNA sequencing data have been deposited in the NCBI Sequence Read Archive (SRA) under BioProject accession number PRJNA1414192 and are publicly available as of the date of publication.

### Functional annotation and pathway enrichment analysis of DEGs

To explore the functional relevance and connectivity of identified DEGs, we performed protein-protein interaction (PPI) analysis using the STRING database (v11.5). Gene symbols corresponding to significant DEGs were uploaded to STRING (https://string-db.org), and interactions were assessed based on evidence from experimental data, curated databases, and text mining. A medium confidence score threshold (≥0.4) was applied to filter interactions. Network visualisation and enrichment analysis (Gene Ontology, KEGG pathways) were performed directly within the STRING web interface. Overlapping genes between the edgeR-derived DEGs and a pre-defined gene set of interest were also identified and annotated to highlight biologically meaningful intersections. These subsets were further analysed for connectivity within the STRING network to identify highly interconnected clusters or modules that may be involved in disease-specific regulatory mechanisms.

To gain biological insights into the differentially expressed genes (DEGs), functional annotation and enrichment analyses were performed using several R/Bioconductor packages, including clusterProfiler, ReactomePA, and DOSE. Gene Ontology (GO) enrichment analysis was carried out across the three major ontologies: Biological Process (BP), Molecular Function (MF), and Cellular Component (CC). KEGG pathway enrichment analysis was performed using the enrichKEGG () function with adjusted *p-value* < 0.05. Similarly, Reactome pathway enrichment was performed using the enrichPathway function from the *ReactomePA* package. All analyses were conducted in R (v4.5).

### Statistical analysis

Experiments were performed in triplicate across three independent biological replicates, and results are presented as mean ± SD. Technical replicates were included solely to assess measurement reproducibility and were averaged prior to statistical analysis. Only independent biological replicates were considered for statistical analyses. Statistical analyses were performed using GraphPad Prism software version 9.0 (GraphPad Software Inc., San Diego, CA, United States). Differences among groups were evaluated using ordinary one-way ANOVA, *p* value <0.05 was considered statistically significant.

## Results

### Characterization of patient-derived organoids of breast cancer

**FIGURE 1:**
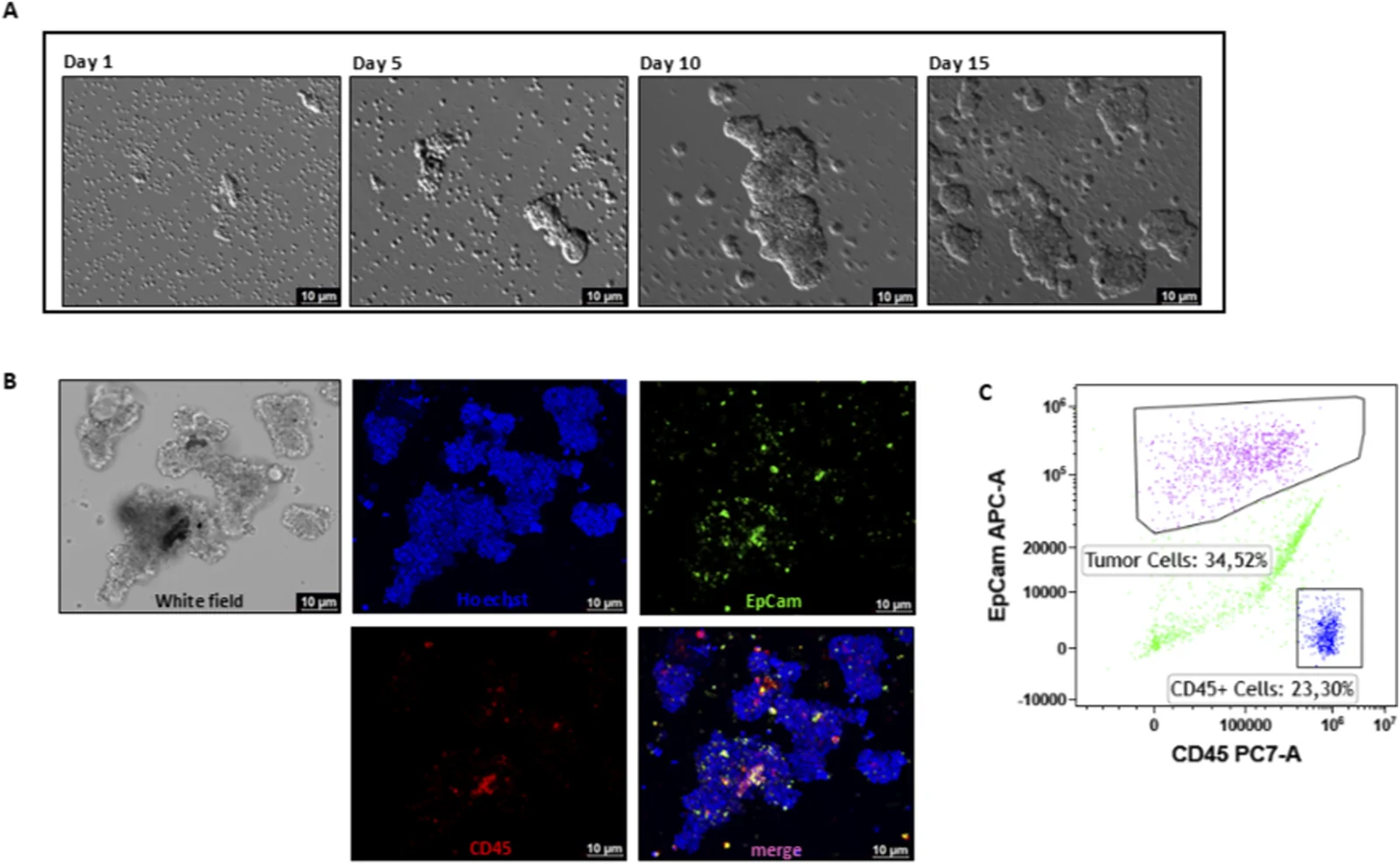
Representative images of *ex vivo* PDO cultures obtained from breast cancer biopsies are shown. Scale bar: 10 µm **(A)**. Characterization of the presence of immune cells in the PDO microenvironment. Maximum projection images of PDOs incubated with α-CD45 (red spots) and α-EpCam (green signal). Cell nuclei were stained with Hoechst 33,342 (blue). Scale bar: 10 µm **(B)**. The collected organoids were analysed using flow cytometry after being stained with EpCam and CD45. Experiments were conducted on all collected samples; the images and plots are representative of one PDO culture **(C)**.

In parallel, we established *ex vivo* 3D breast cancer cultures obtained from surgical samples to estimate the feasibility of *ex vivo* approaches. The human samples were processed by enzymatic digestion to derive primary cell cultures of *ex-vivo* PDOs (Figure 1A). These multicellular organotypic spheroid cultures preserve intercellular interactions and maintain the genetic diversity of the *in vivo* tumor, making them a valid model with which to study cells and the tumor microenvironment. We established 20 out of 25 organoid cultures from biopsy specimens, achieving a successful establishment rate of 80%, which is consistent with published data. One week after seeding in Matrigel, immunofluorescence microscopy confirmed that the tumor/immune cell mixture in all samples was preserved after digestion (Figure 1B). We observed the presence of immune cells in the PDO microenvironment by incubating PDOs with both α-CD45 and α-EpCam and staining the organoid cell nuclei with Hoechst 33,342 (blue signal). We observed colocalization of α-CD45 (red spots) and α-EpCam (green signal). We also evaluated this scenario through flow cytometer analysis to quantify the presence of immune and tumor components, detecting an average cell population formed by CD45^+^ and EpCam + cells, at 23.30% and 34.52%, respectively (Figure 1C).

### Translational machinery and ribosome biogenesis pathways are predominantly upregulated in BC PDOs, with immune pathway trends

Differential expression analysis identified 646 differentially expressed genes meeting the stringent criteria of FDR <0.05 and |logFC| > 0.5 in PDOs generated from breast cancer (BC) compared to non-cancerous (NC) tissues, these last ones used as healthy controls (Supplementary Table S1). Most differentially expressed genes showed up regulation (n = 578, 89.5%), while 68 genes (10.5%) were downregulated. LogFC values ranged from −7.06 to +11.66, with 173 genes (11.6%) demonstrating high statistical significance (FDR <0.005). Functional enrichment analysis using Reactome and KEGG databases revealed distinct pathway signatures associated with the differentially expressed genes. Reactome analysis identified 35 significantly enriched pathways (p.adj <0.05) out of 186 tested pathways (19%) (Supplementary Table S2), while KEGG analysis yielded only one significantly enriched pathway out of 302 analyzed (0.3%).

The most prominently enriched pathways were predominantly associated with translational machinery and ribosome biogenesis, with “Translation” emerging as the top-ranked pathway (24 genes, fold enrichment = 2.71, p. adjust = 4.18 × 10^−3^), followed by “Formation of a pool of free 40S subunits” (13 genes, fold enrichment = 4.20, p.adjust = 4.18 × 10^−3^) and “Eukaryotic Translation Elongation” (12 genes, fold enrichment = 4.21, p.adjust = 4.18 × 10^−3^). Additional translation-related pathways showing significant enrichment included “SRP-dependent cotranslational protein targeting to membrane,” “GTP hydrolysis and joining of the 60S ribosomal subunit,” “Eukaryotic Translation Initiation,” “Cap-dependent Translation Initiation,” “Peptide chain elongation,” and “Eukaryotic Translation Termination” (all p. adjust <0.01). This pronounced enrichment of ribosomal and translational pathways is consistent with the hyperactivated protein synthesis machinery characteristic of cancer cells, which supports uncontrolled proliferation and biomass accumulation. Indeed, dysregulation of ribosome biogenesis has been extensively documented as a hallmark of cancer progression, with cancer cells harboring specialized “onco-ribosomes” that facilitate preferential translation of oncogenes and pro-survival factors, ultimately driving tumor growth and therapeutic resistance (Pelletier et al., 2018; Elhamamsy et al., 2022). Complementing these findings, pathways involved in rRNA processing, including “rRNA processing in the nucleus and cytosol” (18 genes, fold enrichment = 3.04, p.adjust = 4.18 × 10^−3^) and “Major pathway of rRNA processing in the nucleolus and cytosol” (17 genes, fold enrichment = 3.03, p.adjust = 4.43 × 10^−3^), were also significantly enriched, further underscoring the critical role of ribosome biogenesis in sustaining the proliferative capacity of breast cancer organoids.

Beyond translational machinery, our analysis revealed significant enrichment of axon guidance-related signaling pathways, specifically “Regulation of expression of SLITs and ROBOs” (16 genes, fold enrichment = 3.32, p. adjust = 4.18 × 10^−3^) and “Signaling by ROBO receptors” (18 genes, fold enrichment = 2.88, p. adjust = 4.43 × 10^−3^). The SLIT/ROBO signaling axis, originally characterized for its role in neuronal guidance, has emerged as a critical regulator of cancer progression with dual and context-dependent functions in breast malignancies. Multiple studies have demonstrated that SLIT2 and ROBO1 function as tumor suppressors in breast cancer, with their downregulation or loss being associated with enhanced tumor growth, increased metastatic potential, and poor clinical prognosis, particularly in patients developing brain-specific metastases (Qin et al., 2015; Marlow et al., 2008). See Table 3 for an overview of these pathways.

**TABLE 3:** Significantly enriched pathways (p.adjust < 0.05).

Pathway name	Reactome ID	p-value (p.adj)	Gene count	Fold enrich	Pathway description	Gene symbols
Translation	R-HSA-72766	p = 8.55e-06 adj = 0.004	24	2.72	Protein synthesis and ribosome function	MRPS2/EARS2/SRP72/RPL10/RPLP2/MRPS18A/RPL36AL/RPL37/LARS2/MRPL30/MRPS22/RPL21/MRPL34/RPL22/RPL36/RPS13/EEF1B2/MRPL38/RPS24/RPS9/EIF3C/EIF3I/RPL28/SRP14
Regulation of expression of SLITs and ROBOs	R-HSA-9010553	p = 1.75e-05 adj = 0.004	17	3.27	Regulation of SLIT/ROBO axon guidance genes	PSME1/RPL10/PSMA2/PSMB5/PSMB3/RPLP2/RPL36AL/RPL37/RPL21/RPL22/PSMD2/ROBO2/RPL36/RPS13/RPS24/RPS9/RPL28
Eukaryotic translation elongation	R-HSA-156842	p = 2.47e-05 adj = 0.004	12	4.22	Peptide chain elongation during protein synthesis	RPL10/RPLP2/RPL36AL/RPL37/RPL21/RPL22/RPL36/RPS13/EEF1B2/RPS24/RPS9/RPL28
Selenocysteine synthesis	R-HSA-2408557	p = 2.47e-05 adj = 0.004	12	4.22	Selenocysteine incorporation into proteins	PSTK/RPL10/RPLP2/RPL36AL/RPL37/RPL21/RPL22/RPL36/RPS13/RPS24/RPS9/RPL28
rRNA processing in the nucleus and cytosol	R-HSA-8868773	p = 2.53e-05 adj = 0.004	18	3.05	Nuclear and cytosolic rRNA maturation	NOL9/RPL10/NOP10/RPLP2/PDCD11/UTP3/RPL36AL/RPL37/RPL21/RPL22/NOL6/RPL36/ISG20L2/RPS13/RPS24/RPS9/RPL28/RPP25
L13a-mediated translational silencing of Ceruloplasmin expression	R-HSA-156827	p = 3.20e-05 adj = 0.004	13	3.84	Translational silencing by ribosomal protein L13a	RPL10/RPLP2/RPL36AL/RPL37/RPL21/RPL22/RPL36/RPS13/RPS24/RPS9/EIF3C/EIF3I/RPL28
SRP-dependent cotranslational protein targeting to membrane	R-HSA-1799339	p = 3.52e-05 adj = 0.004	13	3.80	SRP-mediated co-translational protein targeting	SRP72/RPL10/RPLP2/RPL36AL/RPL37/RPL21/RPL22/RPL36/RPS13/RPS24/RPS9/RPL28/SRP14
GTP hydrolysis and joining of the 60S ribosomal subunit	R-HSA-72706	p = 3.52e-05 adj = 0.004	13	3.80	60S ribosomal subunit joining	RPL10/RPLP2/RPL36AL/RPL37/RPL21/RPL22/RPL36/RPS13/RPS24/RPS9/EIF3C/EIF3I/RPL28
Signaling by ROBO receptors	R-HSA-376176	p = 3.59e-05 adj = 0.004	19	2.87	ROBO receptor signaling in cell migration	PSME1/RPL10/PFN1/PSMA2/PSMB5/PSMB3/RPLP2/RPL36AL/RPL37/RPL21/RPL22/PSMD2/CAP1/ROBO2/RPL36/RPS13/RPS24/RPS9/RPL28
Major pathway of rRNA processing in the nucleolus and cytosol	R-HSA-6791226	p = 4.49e-05 adj = 0.004	17	3.04	Major rRNA processing pathway in nucleolus	NOL9/RPL10/RPLP2/PDCD11/UTP3/RPL36AL/RPL37/RPL21/RPL22/NOL6/RPL36/ISG20L2/RPS13/RPS24/RPS9/RPL28/RPP25
rRNA processing	R-HSA-72312	p = 4.92e-05 adj = 0.004	18	2.90	Ribosomal RNA processing and maturation	NOL9/RPL10/NOP10/RPLP2/PDCD11/UTP3/RPL36AL/RPL37/RPL21/RPL22/NOL6/RPL36/ISG20L2/RPS13/RPS24/RPS9/RPL28/RPP25
Eukaryotic translation initiation	R-HSA-72613	p = 6.65e-05 adj = 0.004	13	3.58	Translation initiation complex formation	RPL10/RPLP2/RPL36AL/RPL37/RPL21/RPL22/RPL36/RPS13/RPS24/RPS9/EIF3C/EIF3I/RPL28
Cap-dependent translation initiation	R-HSA-72737	p = 6.65e-05 adj = 0.004	13	3.58	Cap-dependent translation initiation	RPL10/RPLP2/RPL36AL/RPL37/RPL21/RPL22/RPL36/RPS13/RPS24/RPS9/EIF3C/EIF3I/RPL28
Peptide chain elongation	R-HSA-156902	p = 8.07e-05 adj = 0.004	11	4.04	Peptide chain elongation during protein synthesis	RPL10/RPLP2/RPL36AL/RPL37/RPL21/RPL22/RPL36/RPS13/RPS24/RPS9/RPL28
Viral mRNA translation	R-HSA-192823	p = 8.07e-05 adj = 0.004	11	4.04	Translation of viral mRNA	RPL10/RPLP2/RPL36AL/RPL37/RPL21/RPL22/RPL36/RPS13/RPS24/RPS9/RPL28
Eukaryotic translation termination	R-HSA-72764	p = 1.20e-04 adj = 0.006	11	3.87	Translation termination and ribosome recycling	RPL10/RPLP2/RPL36AL/RPL37/RPL21/RPL22/RPL36/RPS13/RPS24/RPS9/RPL28
Translation of replicase and assembly of the replication transcription complex	R-HSA-9679504	p = 6.46e-04 adj = 0.025	4	9.45	Viral replicase translation and RTC assembly	CHMP2A/RNF103-CHMP3/PIK3R4/MAP1LC3B
Translation of replicase and assembly of the replication transcription complex	R-HSA-9694676	p = 8.60e-04 adj = 0.028	4	8.82	Viral replicase translation and RTC assembly	CHMP2A/RNF103-CHMP3/PIK3R4/MAP1LC3B

Pathways related to translational machinery, ribosome biogenesis, and axon guidance signaling significantly enriched in breast cancer organoids compared to non-cancerous controls.

Regarding immune- and inflammation-related pathways, the analysis revealed an interesting pattern. While no immune pathways achieved statistical significance after correction for multiple testing (adjusted p-value <0.05), ten immune-related pathways displayed nominal statistical significance (p-value <0.05) (Table 4), including “Antigen processing: Ubiquitin, ATP-independent proteasomal degradation” (p = 0.002, p. adjust = 0.061), “Antigen processing-Cross presentation” (p = 0.003, p. adjust = 0.067), “Interleukin-1 family signaling” (p = 0.010, p. adjust = 0.147), “MHC class II antigen presentation” (p = 0.013, p. adjust = 0.160), and “Toll-like Receptor Cascades” (p = 0.037, p.adjust = 0.272). These findings suggest a trend toward immune pathway enrichment that did not survive stringent multiple testing correction, potentially reflecting residual expression of immune-related genes in the epithelial compartment or low-level representation of these pathways in the organoid transcriptome. In contrast, neurotrophin signaling pathways showed no enrichment whatsoever, with no pathways achieving even nominal statistical significance (p-value <0.05). This complete absence of neurotrophin-related pathways may reflect the organoid culture systems, which typically lack stromal, neural, immune, and vascular components that constitute the tumor microenvironment *in vivo*. While patient-derived organoids faithfully preserve the epithelial architecture, genetic alterations, and transcriptomic signatures of primary tumors, they do not fully recapitulate the complex interactions between tumor cells and infiltrating immune cells, cancer-associated fibroblasts, or endothelial cells (Saeki et al., 2023; LeSavage et al., 2022).

**TABLE 4:** Immune/Inflammation pathways with nominal significance.

Pathway name	Reactome ID	p-value (p.adj)	Gene count	Fold enrich	Pathway description	Gene symbols
Antigen processing-Cross presentation	R-HSA-1236975	p = 0.004 adj = 0.082	9	2.83	MHC-I cross-presentation of exogenous antigens	CYBA/PSME1/PSMA2/TLR2/PSMB5/PSMB3/CTSS/SEC22B/PSMD2
Ubiquitin mediated degradation of phosphorylated Cdc25A	R-HSA-69601	p = 0.005 adj = 0.082	6	3.82	Proteasomal degradation pathway (involved in antigen processing)	PSME1/PSMA2/PSMB5/PSMB3/CDC25A/PSMD2
Interleukin-1 family signaling	R-HSA-446652	p = 0.007 adj = 0.101	11	2.38	IL-1 family cytokine signaling (IL-1α/β, IL-18, IL-33)	IL18BP/IL1RN/PSME1/IL1RAP/PSMA2/PSMB5/PSMB3/IRAK1/IL18RAP/PSMD2/SKP1
Interleukin-1 signaling	R-HSA-9020702	p = 0.008 adj = 0.119	9	2.59	IL-1α/β pro-inflammatory receptor signaling	IL1RN/PSME1/IL1RAP/PSMA2/PSMB5/PSMB3/IRAK1/PSMD2/SKP1
MHC class II antigen presentation	R-HSA-2132295	p = 0.012 adj = 0.154	9	2.42	MHC-II antigen presentation to CD4^+^ T cells	HLA-DQA1/TUBA1C/LGMN/ACTR1A/CTSS/DCTN1/TUBA1B/TUBA1A/DNM1
Activation of NF-kappaB in B cells	R-HSA-1169091	p = 0.016 adj = 0.177	6	2.96	NF-κB activation in B lymphocytes	PSME1/PSMA2/PSMB5/PSMB3/PSMD2/SKP1
Cross-presentation of soluble exogenous antigens (endosomes)	R-HSA-1236978	p = 0.016 adj = 0.177	5	3.37	Endosomal cross-presentation of soluble antigens	PSME1/PSMA2/PSMB5/PSMB3/PSMD2
Toll-like receptor cascades	R-HSA-168898	p = 0.036 adj = 0.240	10	1.92	Innate immune pattern recognition signaling	ITGB2/PIK3R4/LGMN/TLR2/IRAK1/CTSS/RPS6KA5/SKP1/TRAF3/DNM1
Downstream signaling events of B Cell receptor (BCR)	R-HSA-1168372	p = 0.039 adj = 0.247	6	2.39	BCR downstream signaling cascade	PSME1/PSMA2/PSMB5/PSMB3/PSMD2/SKP1
Class I MHC mediated antigen processing and presentation	R-HSA-983169	p = 0.041 adj = 0.253	18	1.56	MHC-I antigen processing and presentation	TRIM21/CYBA/PSME1/LRRC41/RNF34/PSMA2/PIK3R4/TLR2/SIAH1/PSMB5/DCAF1/PSMB3/UBR1/CTSS/GPR75-ASB3/SEC22B/PSMD2/SKP1

Immune and inflammation-related pathways showing nominal statistical significance (p < 0.05) without surviving multiple testing correction (p.adjust ≥ 0.05). These pathways suggest immune-related transcriptional activity in breast cancer organoids that may reflect residual immune gene expression in the epithelial compartment.

### PPI network analysis identifies a direct IL1RAP–NTRK3 interaction bridging inflammatory and neurotrophin signaling

We performed protein-protein interaction network analysis using the STRING database, focusing initially on IL1RAP (Interleukin 1 Receptor Accessory Protein), a key component identified in the nominally enriched “Interleukin-1 family signaling” pathway (10 genes, p = 0.010) and included in our 173 most differentially expressed genes (FDR < 0.005). IL1RAP is a critical co-receptor for both IL-1α and IL-1β signaling, forming a heterodimeric complex with IL-1R1 that is essential for downstream inflammatory signaling cascade activation, including NF-κB and MAPK pathway stimulation (Davies et al., 2010). The selection of IL1RAP as a seed gene for network expansion was motivated by its central role in orchestrating interleukin-1-mediated inflammatory responses and by emerging evidence that suggest potential crosstalk between inflammatory cytokine signaling and neurotrophic factor pathways in cancer progression (Huang et al., 2025).

In the context of breast cancer, such inflammatory-neurotrophin crosstalk has been implicated in tumor progression, with IL-1 signaling promoting an aggressive phenotype while neurotrophins such as NGF (Nerve Growth Factor) and BDNF (Brain-Derived Neurotrophic Factor) have been associated with tumor cell survival, invasion, and metastasis through activation of their cognate Trk receptors (Patani et al., 2011; Dollé et al., 2003). Notably, BDNF expression in breast cancer has been correlated with adverse pathological features, including nodal positivity, reduced disease-free survival, and poor overall survival (Patani et al., 2011), while cancer-derived neurotrophins have been shown to drive neurite growth and establish reciprocal signaling between cancer cells and nerve endings, facilitating tumor expansion and metastatic dissemination (Zahalka and Frenette, 2020). Therefore, identifying potential interaction networks linking IL1RAP-mediated inflammatory signaling to neurotrophin-associated genes could provide mechanistic insights into how these systems coordinate to influence breast cancer organoid biology.

We performed protein-protein interaction network (Supplementary Table S3) using the entire set of approximately 600 differentially expressed genes identified in our breast cancer organoid model. We then focused our analysis on IL1RAP-centered interaction networks to investigate whether this key inflammatory mediator exhibits connectivity with neurotrophin-related genes. This network-based approach revealed that IL1RAP is directly connected to six DEG genes: IL1RN (Interleukin-1 Receptor Antagonist), IL18RAP (Interleukin-18 Receptor Accessory Protein), IL18BP (Interleukin-18 Binding Protein), TLR2 (Toll-like Receptor 2), IRAK1 (Interleukin-1 Receptor-Associated Kinase 1), and NTRK3 (Neurotrophic Receptor Tyrosine Kinase 3/TrkC). Notably, the presence of NTRK3/TrkC—a high-affinity receptor for neurotrophin-3 (NT-3) that mediates neuronal survival and differentiation signals through activation of PI3K/AKT and RAS/MAPK pathways—within the IL1RAP interaction network provides hypothesis-generating computational basis for investigating potential crosstalk between interleukin-1 inflammatory signaling and neurotrophin pathways; experimental validation of this association is required to establish whether it reflects a direct biochemical interaction in breast cancer cells (Jin, 2020; Cocco et al., 2018). In breast cancer, NTRK3 expression has been associated with tumor biology and patient prognosis, with low NTRK3 expression correlating with poorer outcomes and alterations in tumor-infiltrating immune cell populations (Choi et al., 2025). Given the significant downregulation of NTRK3 (logFC = −4.92, FDR = 0.019) and its central role in neurotrophin signaling, we performed targeted network expansion to identify first-degree protein-protein interactions of NTRK3 with a combined score >0.5. In building the network we also considered the 20 proteins belonging to the same reactome pathway (R-HSA-388844.4: Receptor-type tyrosine-protein phosphatases) as NTRK3 even if they were not present in our list of deregulated genes. This results in the selection of eight genes: IL1RAPL1, PTPRD, PTPRS, PTPRF, IRF2BP2, SLITRK3, SLITRK1 and BDNF genes (only IRF2BP2 and BDNF are also in DEGs list). We prioritized interactors and potential non-classical regulatory functions selecting SLITRK3 (SLIT and NTRK-like family member 3) and IRF2BP2 (interferon regulatory factor 2 binding protein 2) for further investigation in organoid model (Meng et al., 2019). SLITRK3 was selected based on its demonstrated physical interaction with NTRK3 and ability to modulate ligand-dependent NTRK3 activation in cancer cells (Bollig-Fischer et al., 2021). Although SLITRK3 shares structural homology with neurotrophin receptors, it is not formally annotated in canonical neurotrophin signaling pathways but functions as a regulatory co-partner of NTRK receptors. IRF2BP2 was selected based on its high betweenness centrality in the NTRK3 interaction network (STRING combined score >0.5) and its established role as a transcriptional corepressor in interferon and cytokine signaling pathways, suggesting potential crosstalk between oncogenic receptor signaling and immune modulation (Pastor et al., 2021).

The inclusion of these interactors aimed to explore regulatory mechanisms beyond classical neurotrophin-TRK signaling and to investigate the potential convergence of oncogenic and immune-regulatory networks in breast cancer organoids.

**FIGURE 2:**
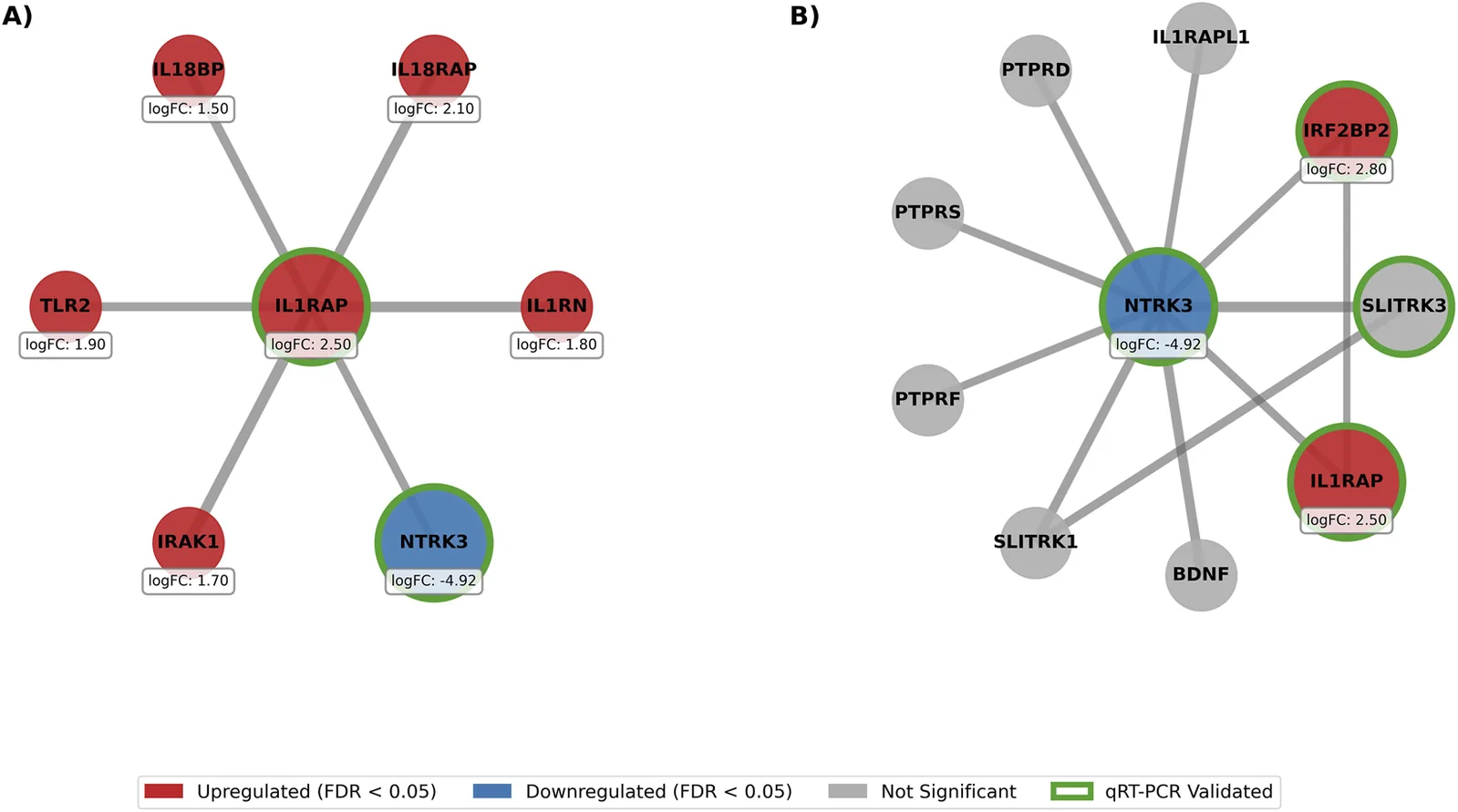
Protein-protein interaction networks centered on IL1RAP and NTRK3 in breast cancer patient-derived organoids. **(A)** IL1RAP-centered inflammatory network showing direct protein-protein interactions with key inflammatory signaling components including IL1RN (Interleukin-1 Receptor Antagonist), IL18RAP (Interleukin-18 Receptor Accessory Protein), IL18BP (Interleukin-18 Binding Protein), TLR2 (Toll-like Receptor 2), IRAK1 (Interleukin-1 Receptor-Associated Kinase 1), and NTRK3 (Neurotrophic Receptor Tyrosine Kinase 3). **(B)** NTRK3-centered network displaying neurotrophin-immune crosstalk through interactions with SLITRK3 (SLIT and NTRK-like family member 3), IRF2BP2 (Interferon Regulatory Factor 2 Binding Protein 2), neurotrophin pathway components (BDNF, SLITRK1), phosphatases (PTPRD, PTPRS, PTPRF), and IL1RAP. Node colors indicate differential expression status: red nodes represent significantly upregulated genes (FDR <0.05), blue nodes represent significantly downregulated genes (FDR <0.05), and gray nodes indicate genes without significant differential expression. Genes with green borders (IL1RAP, NTRK3, SLITRK3, IRF2BP2) were validated by qRT-PCR. Expression values (logFC) are shown for significantly differentially expressed genes. Edge colors represent the type of evidence supporting each interaction: green edges indicate experimental evidence, purple edges represent curated database evidence, orange edges show text-mining support, and brown edges indicate co-expression relationships. Edge width is proportional to the STRING combined interaction confidence score (range: 0.5–0.95), with thicker edges representing higher confidence interactions.

Based on this analysis, we selected four genes for experimental validation in organoids by qRT-PCR: IL1RAP and IRF2BP2 (significantly upregulated DEGs with immune-regulatory functions), SLITRK3 (not significantly deregulated in RNA-seq analysis), and NTRK3 (significantly downregulated at gene-level in RNA-seq, logFC = −4.92, FDR = 0.019), the latter retained specifically because of its role as the direct network bridge between IL1RAP-mediated inflammatory signaling and the neurotrophin axis (Figure 2). It should be noted that, considering the transcript-level analysis, NTRK3 remains an ambiguous biomarker in the current study (downregulated in RNA-seq and upregulated *in vitro* analyses).

It should be noted that NTRK3 was retained for validation despite its net downregulation in the RNA-seq analysis, given its central role as the direct network bridge between inflammatory and neurotrophin signaling. As discussed below, the direction of NTRK3 expression in qRT-PCR validation showed apparent discordance with the RNA-seq result, a finding that requires careful interpretation in light of technical and biological considerations specific to this gene.

### SLITRK3, IL1RAP and IRF2BP2 are consistently upregulated in BC PDOs and associated with elevated IL-1β and IFN-γ secretion

**FIGURE 3:**
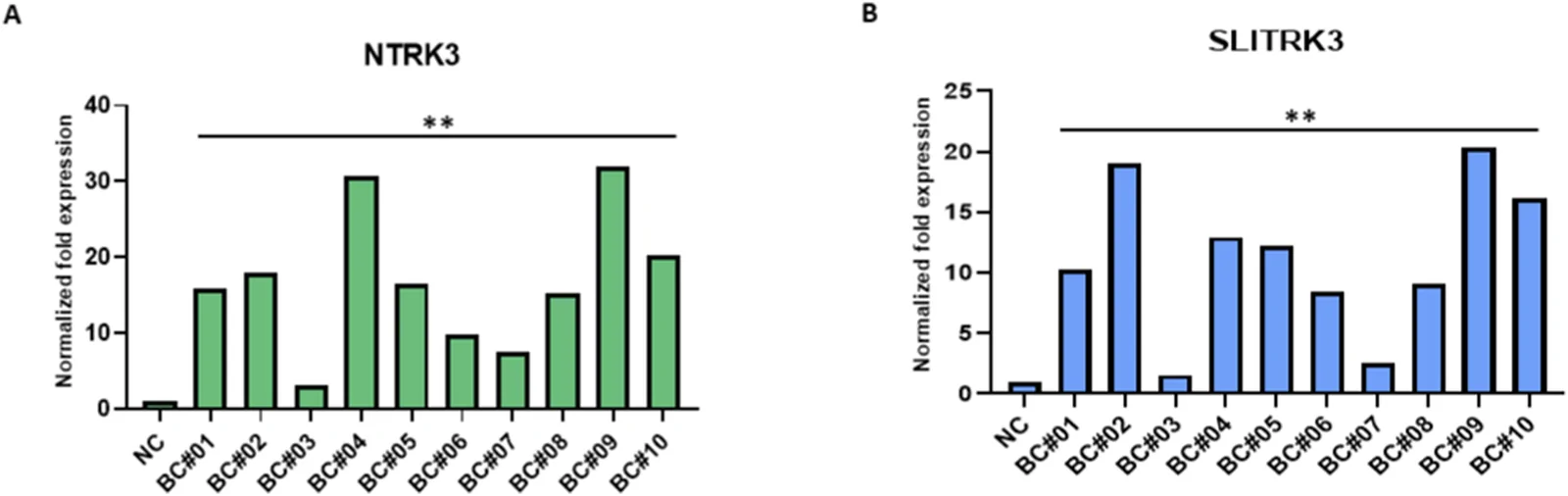
Characterization of NTRK3 and SLITRK3 in tumor and non-cancerous PDOs. Real time qPCR analysis of NTRK3 **(A)** and SLITRK3 **(B)** in PDOs derived from tumor breast tissue (BC#1-#10) vs. PDOs from fibroadenoma samples considered as non-cancerous tissue sample (NC). Results were normalized to 18S mRNA and analyzed by ΔCt method. One way ANOVA test followed by Tukey’s test were used for statistical analysis. **p < 0.01.

Within the multifaceted landscape of breast cancer biology, the coordinated regulation of oncogenic, inflammatory, and immune-related pathways plays a crucial role in shaping tumor behavior and progression. In this context, we investigated the expression and potential functional interplay of four genes NTRK3, SLITRK3, IL1RAP, and IRF2BP2- which are implicated in diverse biological processes including tumor cell signaling, stemness maintenance, and immune modulation. These genes were selected based on their reported or putative roles in cancer-related pathways, particularly in the context of inflammation and immune signaling. To assess differential gene expression, we performed quantitative real-time PCR (qRT-PCR) on a panel of breast cancer patient-derived organoids (BC PDOs, samples #01–#10) and compared the results to organoids generated from non-cancerous breast tissue (NC PDOs), serving as healthy controls. The analysis revealed a consistent and statistically significant upregulation of SLITRK3, IL1RAP, and IRF2BP2 in BC PDOs compared to controls (p < 0.05 for all comparisons) (Figures 3A,B). NTRK3 also showed higher qRT-PCR signal in BC PDOs; however, this result cannot be unambiguously interpreted as validated upregulation given the discordance with the RNA-seq gene-level result (logFC = −4.92, FDR = 0.019) and the findings of the transcript-level analysis (see below). The elevated expressions of SLITRK3, IL1RAP, and IRF2BP2 suggest that these genes are actively involved in oncogenic processes specific to the tumor environment.

**FIGURE 4:**
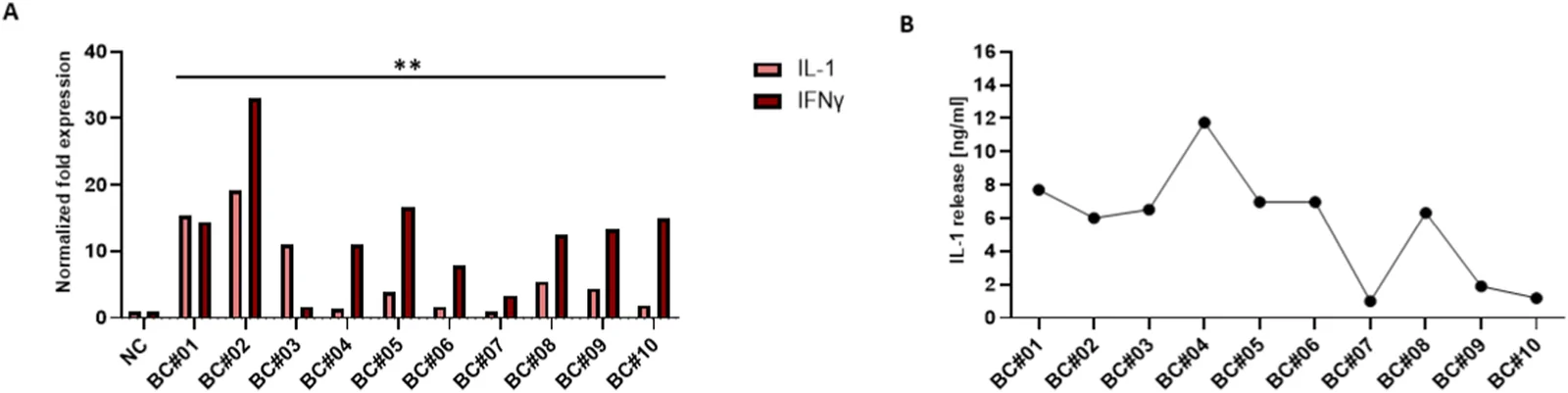
Activation of key inflammatory pathways within PDOs microenvironment. **(A)** Real time qPCR analysis of IL-1 and IFN-γ in PDOs derived from tumor breast tissue (BC#1-#10) vs. PDOs from fibroadenoma samples considered as non-cancerous tissue sample (NC). Results were normalized to 18S mRNA and analyzed by ΔCt method. One way ANOVA test followed by Tukey’s test were used for statistical analysis. **p < 0.01. **(B)** Quantification of IL-1 release by breast cancer PDOs (BC#1-#10) measured by ELISA assay (ng/ml). Statistical analysis was performed using ANOVA test followed by Tukey’s test.

In parallel, we evaluated the expression levels of pro-inflammatory cytokines, focusing on interleukin-1 (IL-1) and interferon-gamma (IFN-γ), which are known to engage the IL1RAP and IRF2BP2 signaling axes, respectively. The qRT-PCR data demonstrated a marked increase in both IL-1 and IFN-γ mRNA levels in the BC PDOs, indicating activation of key inflammatory pathways within the tumor-derived organoid microenvironment (Figure 4A). To determine whether the transcriptional upregulation of IL1RAP was associated with functional cytokine signaling, we conducted an ELISA-based quantification of soluble IL-1 in the culture supernatants (Figure 4B). The results showed significantly elevated levels of IL-1 protein in the media collected from BC PDOs compared to NC PDOs, thereby confirming the presence of an active pro-inflammatory milieu.

## Discussion

The molecular characterization of patient-derived organoids (PDOs) through high-throughput transcriptomic profiling has provided valuable and novel insights into gene expression patterns associated with the various molecular subtypes of breast cancer, including luminal A, luminal B, HER2-enriched, and triple-negative breast cancer (TNBC). These organoid-based models retain much of the intra- and intertumoral heterogeneity of the original tumors, making them an ideal system to investigate subtype-specific transcriptional programs, oncogenic signaling pathways, and potential biomarkers of disease progression and therapeutic response.

Preliminary we evaluated the presence of immune cells in the PDO microenvironment, and we observed a colocalization of CD45 and EpCam; in particular, CD45^+^ was used as marker to ensure the presence of immune cells, while EpCam represents a diagnostic marker for the detection of carcinoma cells because it is expressed by normal and malignant epithelia. This result was validated by FACS analysis confirming that phenotypic characteristics of the different tumor components are preserved.

The integration of immune profiling with transcriptomic data in PDOs offers an unprecedented opportunity to explore the dynamic crosstalk between tumor cells and the immune microenvironment in a patient-specific context. In this study, we investigated the transcriptional and functional profiles of breast cancer patient-derived organoids (PDOs), focusing on the interplay between oncogenic signaling and immune-related pathways.

Our results revealed a significant upregulation of SLITRK3, IL1RAP, and IRF2BP2 in breast cancer patient-derived organoids (PDOs) compared to organoids derived from non-tumoral breast tissue. To address the apparent upregulation of NTRK3 by qRT-PCR that contrasts with its significant downregulation in the RNA-seq DEG analysis, we performed a *post hoc* transcript-level analysis of NTRK3 followed by DTE (DESeq2) and DTU (DRIMSeq + stageR) testing. This analysis showed that the predominant NTRK3 signal in NE PDOs derives from multiple isoforms (NTRK3-203, −209, −205), while the apparent BC signal is attributable to a single isoform (NTRK3-202). Furthermore, *in silico* PCR confirmed that the qRT-PCR primers used in this study amplify eight NTRK3 isoforms simultaneously, including those that are NE-dominant; the primers are therefore not isoform-specific. These combined findings indicate that the discordance between RNA-seq and qRT-PCR for NTRK3 cannot be resolved with the available data. We therefore classify NTRK3 as a network hub of interest (directly bridging IL1RAP-mediated inflammatory signalling and the neurotrophin axis through a confirmed protein–protein interaction) rather than a validated upregulated biomarker. The validated upregulated biomarkers of this study are IL1RAP, IRF2BP2, and SLITRK3. The IL1RAP–NTRK3 interaction remains a biologically motivated and structurally supported finding that warrants investigation with isoform-resolved sequencing approaches and protein-level validation in future work.

The confirmed transcriptional signature was accompanied by elevated expression of pro-inflammatory cytokines IL-1 and IFN-γ, suggesting a coordinated activation of tumor-intrinsic and immune-modulatory mechanisms.

The upregulation of SLITRK3 supports previous findings implicating the neurotrophin signalling pathway in breast cancer progression and maintenance of stem-like properties (Bollig-Fischer et al., 2021). NTRK3, while not validated as upregulated in the current dataset, remains relevant as the direct network interface between IL1RAP-mediated inflammatory signalling and the neurotrophin pathway.

NTRK3, in particular, has been associated with both oncogenic fusion proteins and survival signaling in various cancers, while SLITRK family members are emerging as modulators of cellular adhesion and proliferation in the tumor context (Wang et al., 2015). Their co-upregulation may reflect an adaptive advantage in supporting tumor growth or metastasis, particularly in aggressive breast cancer subtypes. Importantly, our data highlight IL1RAP and IRF2BP2 as potential biomarkers associated with the tumor immune microenvironment within the tumor organoid system.

IL1RAP, a co-receptor for IL-1 receptor signaling, has been increasingly recognized as a key mediator of chronic inflammation in cancer, and its elevated expression in our BC PDOs -confirmed at both transcript and protein levels-points to a functionally active IL-1 signaling axis. At the same time, the upregulation of IRF2BP2 (a transcriptional corepressor involved in interferon and cytokine signalling) and increased IFN-γ expression has been reported as potentially reflecting a complex immunomodulatory environment. This environment may be associated with mechanisms of immune-mediator or reduced sensitivity to immune surveillance (Castro et al., 2018).

The functional relevance of these pathways was further substantiated by our ELISA data, demonstrating increased secretion of soluble IL-1 in BC PDO cultures. This not only validates the transcriptional data but also provides insight into the secretory phenotype of the tumor organoids, potentially mimicking the inflammatory microenvironment of *in vivo* breast tumors. From a translational perspective, these findings underscore the potential of targeting IL1RAP and associated cytokine pathways as a therapeutic strategy, especially in breast cancers characterized by heightened inflammatory signaling (Zheng et al., 2018). IL-1 blockade has already shown promise in clinical settings for other cancers, and its exploration in breast cancer, particularly in conjunction with immune checkpoint inhibition or anti- SLITRK3 therapies, could offer synergistic benefits. Moreover, the use of PDOs in this study highlights their relevance as patient-specific platforms for studying tumor biology, immune interactions, and therapeutic response. The retention of molecular and functional characteristics observed in the PDOs supports their utility not only as preclinical models but also as tools for precision medicine. In conclusion, our results point to a coordinated upregulation of oncogenic, inflammatory, and immune-regulatory genes in breast cancer PDOs. These data expand our understanding of the molecular complexity of breast cancer and suggest new avenues for biomarker discovery and candidate therapeutic target, particularly within the inflammatory signaling axis.

In conclusion, our study provides an organoid-derived computational framework for risk stratification rather than only a list of candidate genes. At the transcriptome-wide level, the dominant biological signal is the hyperactivation of translational machinery and ribosome biogenesis, consistent with the pro-proliferative state of cancer cells. Moreover, immune-related pathways showed an interesting biologically trends. The inflammatory–neurotrophin connections are therefore an interesting network-derived result, supported by the direct PPI link between IL1RAP and NTRK3 and confirmed by targeted qRT-PCR and ELISA validation, rather than primary enrichment findings. IL1RAP and IRF2BP2 emerge as candidate regulatory nodes warranting further functional investigation. Using PDOs enabled us to preserve the epithelial architecture of the tumour and apply an integrative analytical strategy that revealed biologically relevant gene interactions. This integrative strategy reinforces the translational value of PDO-based research and provides a data-supported foundation for future mechanistic studies and hypothesis-driven clinical investigation.

## Data Availability

The datasets presented in this study can be found in online repositories. The names of the repository/repositories and accession number(s) can be found in the article/Supplementary Material.
