# Supplementary material for: Integrated transcriptomic and immune-associated network analysis of breast cancer patient-derived organoids reveals candidate inflammatory biomarkers
Source: Front Bioeng Biotechnol. 2026 Aug 28;14:1900123. doi: 10.3389/fbioe.2026.1900123 (PMC13562241; doi:10.3389/fbioe.2026.1900123)
Supplement: Supplementary file 1 [file Table4.docx]

**Supplementary Table 4. Clinicopathological characteristics of the selected patients**

| **Patient ID** | **E-cadherin** | **ER (%)** | **PgR (%)** | **Ki-67 (%)** | **HER2 (Hercep test, score)** |
| --- | --- | --- | --- | --- | --- |
| #01 | NA | 90 | 35 | 18 | Negative (score 0) |
| #02 | NA | 90 | 15 | 35 | Negative (score 1+) |
| #03 | Positive | 99 | 99 | 20 | Negative (score 0) |
| #04 | Negative | 95 | Negative | 25 | Negative (score 1+) |
| #06 | NA | 90 | 70 | 30 | Negative (score 1+) |
| #07 | Positive | 95 | 55 | 20 | Negative (score 0) |
| B#17 | NA | 95 | 5 | 22 | Negative (score 1+) |
| #18 | NA | 95 | Negative | 15 | Negative (score 1+) |
| #19 | Positive | 85 | 90 | 20 | Negative (score 0) |
| #20 | Positive | 95 | 90 | 40 | Negative (score 0) |

*ER: estrogen receptor; PgR: progesterone receptor; NA: not assessed.*
